# Challenges in treatment of a patient suffering from neuroendocrine tumor G1 of the hilar bile duct: a case report

**DOI:** 10.1186/s12876-021-02019-6

**Published:** 2022-01-08

**Authors:** Biao Zhang, Shuang Li, Zhen Sun, Xu Chen, Bing Qi, Qingkai Zhang, Guixin Zhang, Dong Shang

**Affiliations:** 1 Department of General Surgery, Clinical Laboratory of Integrative Medicine, The First Affiliated Hospital of Dalian Medical University, Dalian, 116011 Liaoning China; 2 Institute of Integrative Medicine, Dalian Medical University, Dalian, 116044 Liaoning China; 3 Department of General Surgery, The Second Hospital of Jilin University, Changchun, 130022 Jilin China

**Keywords:** Biliary neuroendocrine tumors, Diagnosis, Treatment, R1 resection, Case report

## Abstract

**Background:**

Neuroendocrine tumors (NETs) arise from neuroendocrine cells and are extremely rare in the biliary tract. Currently, there are no guidelines for the diagnosis and treatment of biliary NETs. We presented a case with NETs G1 of the hilar bile duct and the challenges for her treatment.

**Case presentation:**

A 24-year-old woman was presented to our department with painless jaundice and pruritus, and the preoperative diagnosis was Bismuth type II hilar cholangiocarcinoma. She underwent Roux-en-Y hepaticojejunostomy with excision of the extrahepatic biliary tree and radical lymphadenectomy. Unexpectedly, postoperative pathological and immunohistochemical examination indicated a perihilar bile duct NETs G1 with the microscopic invasion of the resected right hepatic duct. Then the patient received 3 cycles of adjuvant chemotherapy (Gemcitabine and tegafur-gimeracil-oteracil potassium capsule). At present, this patient has been following up for 24 months without recurrence or disease progression.

**Conclusion:**

We know little of biliary NETs because of its rarity. There are currently no guidelines for the diagnosis and treatment of biliary NETs. We reported a case of perihilar bile duct NETs G1 with R1 resection, as far as we know this is the first report. More information about biliary NETs should be registered.

## Background

Neuroendocrine tumors (NETs) are originated from the neuroendocrine cell system and have a steadily increased incidence from 1.09/100000 in 1973 to 6.98/100000 in 2012 [1, 2]. NETs mainly occur in gastrointestinal tract (45.2%), respiratory system (30.2%) and pancreas (15.3%) [3]. The incidence of extrahepatic biliary neuroendocrine tumors (EBNETs) is extremely low and only accounts for 0.2–2% of all gastrointestinal NETs [4]. The most familiar locations of EBNETs are found in the common hepatic duct and the distal common bile duct (19.2%), followed by the middle of the common bile duct (17.9%), the cystic duct (16.7%), and the proximal common bile duct (11.5%) [5]. NETs are histologically graded into well differentiated (grade 1, 2, or 3 NETs) or poorly differentiated (neuroendocrine carcinomas) tumors. Here we reported a case of perihilar bile duct NETs G1.

## Case presentation

**Fig. 1:**
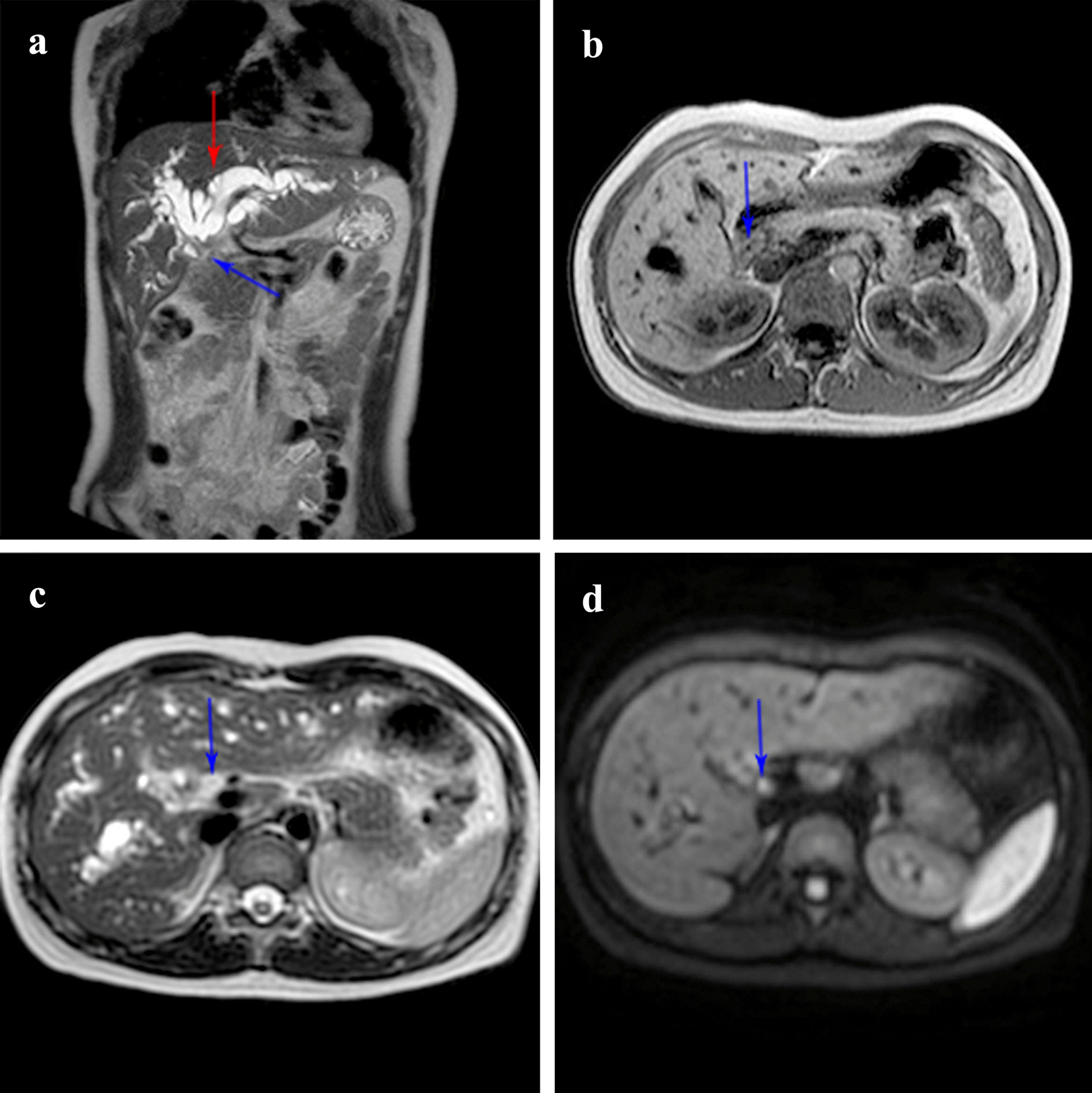
Preoperative abdominal MRI. **a** Magnetic resonance cholangiopancreatography (MRCP) showed that the tumor was located near the bifurcation of the hepatic duct (blue arrow) with diffuse intrahepatic bile duct dilation (red arrow). **b** T1-weighted image (T1WI) showed that the tumor had lower signal intensity (SI) than the hepatic parenchyma (blue arrow). **c** T2-weighted image (T2WI) showed that the tumor had higher SI than the hepatic parenchyma (blue arrow). **d** Diffusion-weighted image (DWI) showed the tumor had higher SI than the hepatic parenchyma (blue arrow)

**Fig. 2:**
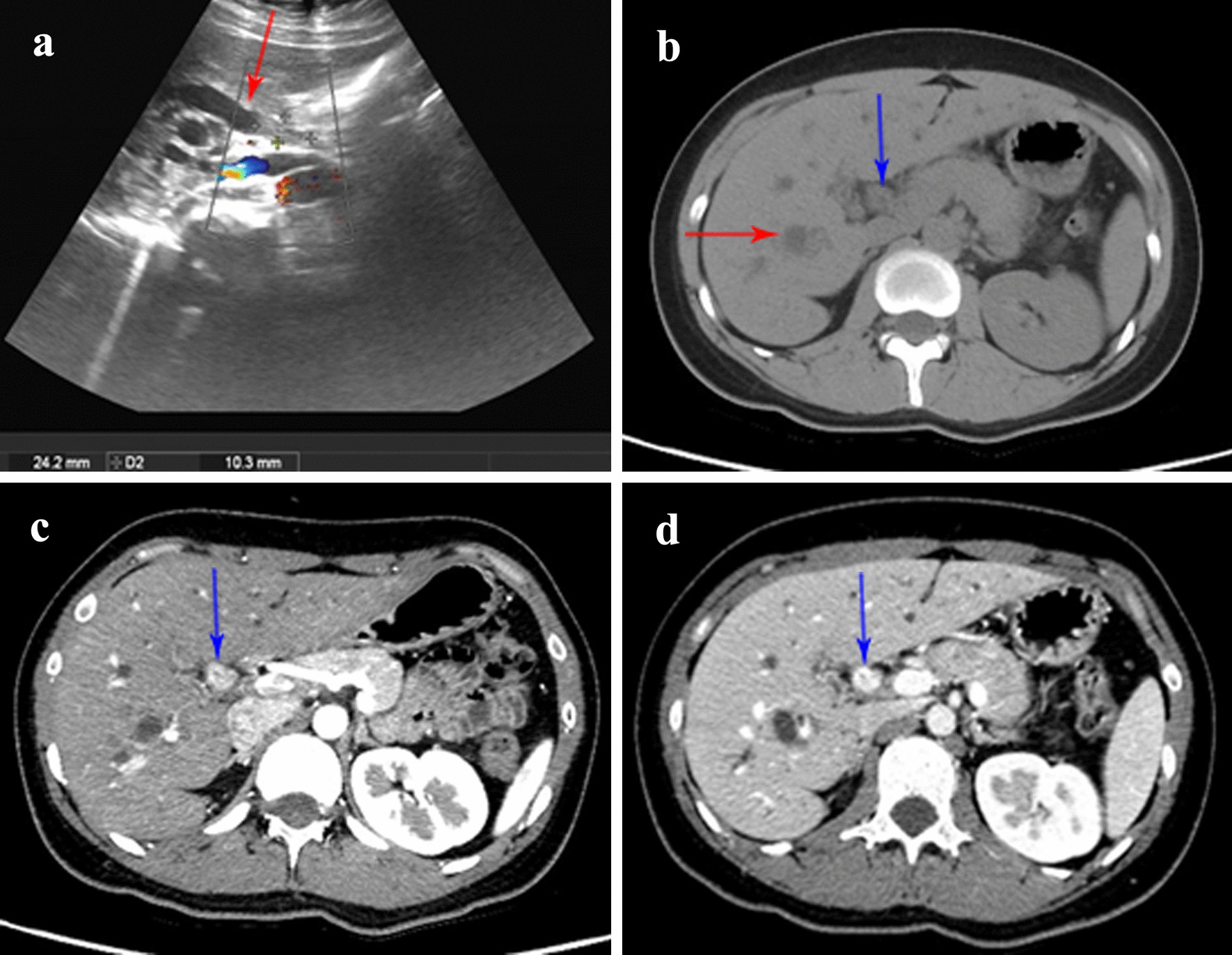
Preoperative abdominal ultrasonography and CT. **a** Abdominal ultrasonography indicated the dilation of bile duct (red arrow). **b** Non-enhanced phase of CT showed that the intrahepatic bile duct was marked dilated (red arrow) and the tumor was located in the common hepatic duct (blue arrow). **c** Arterial-phase of CT showed the tumor was of higher density than liver parenchyma (blue arrow). **d** Portal-venous phase of CT showed the tumor was of higher density was of higher density than liver parenchyma (blue arrow)

A 24-year-old woman was presented to our department with painless jaundice and pruritus for 6 days. Magnetic resonance imaging (MRI) in a local hospital indicated that biliary obstruction at the hepatic hilus and highly suspected hilar cholangiocarcinoma (Fig. 1). The patient suffered yellow skin, itching all over the body, dark urine, and light-colored stool. The patient had neither abdominal tenderness nor a palpable mass in the right upper quadrant of the abdomen. The patient had no family history of cancer or hepatobiliary disease. The laboratory examinations showed the following: alanine aminotransferase, 22 IU/L (normal, 7–40 IU/L); Aspartate transaminase, 27 IU/L (normal, 13–35 IU/L); Total bilirubin, 193.1 umol/L (normal, ≤ 23.0 umol/L); Direct bilirubin, 153.5 umol/L (normal, ≤ 7.0 umol/L); Alkaline phosphatase, 231 IU/L (normal, 35–100 IU/L); Gamma-glutamyltransferase, 94 IU/L (normal, 7–45 IU/L). Tumor markers were within normal limits, carcinoembryonic antigen (CEA), 1.44 ng/mL (normal, 0–5 ng/mL); alpha-fetoprotein (AFP), 1.91 IU/L (normal, 0–5.8 IU/L); carbohydrate antigen 19-9 (CA-19-9), 20.7 IU/L (normal, 0–27 IU/L); CA-125, 14.6 IU/L (normal, 0–35 IU/L). Abdominal ultrasonography examination showed dilation of the hepatic bile duct (Fig. 2). Abdominal contrast-enhanced computed tomography (CT) showed that the intrahepatic bile duct was marked dilated, the tumor was located at the common hepatic duct with a higher density than liver parenchyma in arterial-phase and portal-venous phase (Fig. 2).

**Fig. 3:**
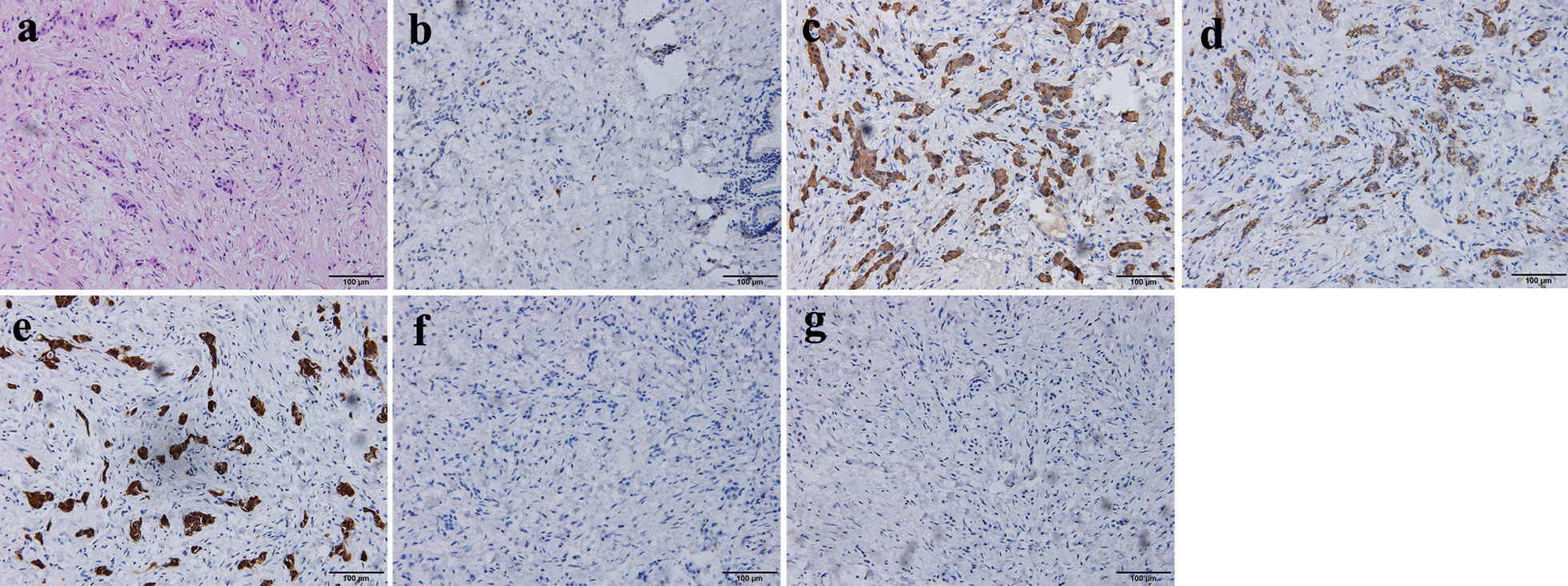
Postoperative pathological and immunohistochemical examination. Hematoxylin–eosin staining showed that tumor cells grew in infiltrating glandular ducts and nests. Heterotypic cells were cubic, with round, dark-stained nuclei, acidophilic and abundant cytoplasm, and proliferation of surrounding fibrous tissue (**a**, ×200). Immunohistochemical examination showed Ki-67 < 2% (**b**, ×200), the positivity for CgA (**c**, ×200), CD56 (**d**, ×200) and Syn (**e**, ×200), the negativity for CK-7 (**f**, ×200) and CK-20 (**g**, ×200)

**Fig. 4:**
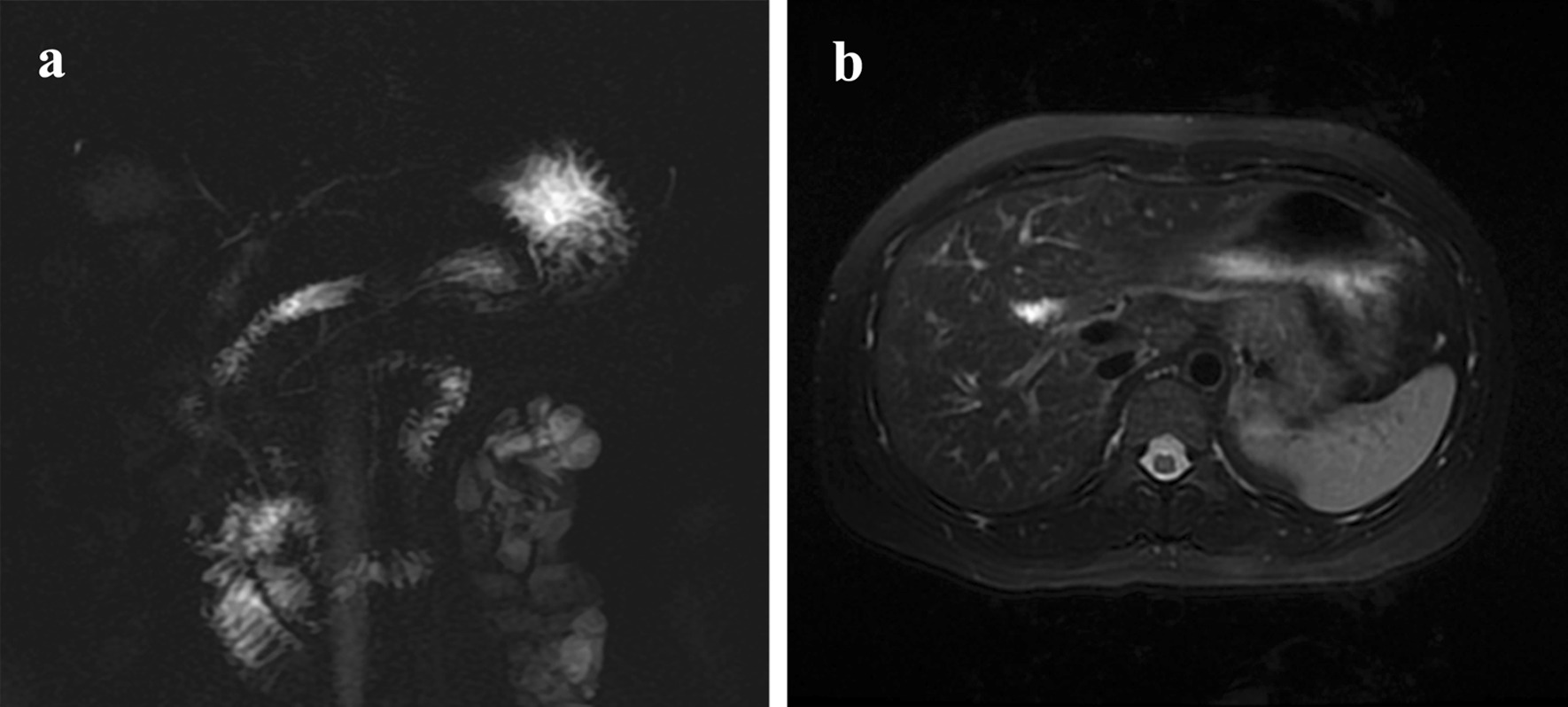
Abdominal MRI of postoperative follow-up. **a** MRCP indicated that the bile-intestinal anastomosis were unobstructed, the intrahepatic bile duct was not dilated, and there’s no recurrence of the disease. **b** T2WI indicated that there’s no recurrence of the disease

Subsequently, percutaneous transhepatic biliary drainage (PTBD) was performed to reduce jaundice and improve liver function, then the patient was referred for operation with the preoperative diagnosis of Bismuth type II hilar cholangiocarcinoma. At surgery, we detected a nodular mass in the perihilar bile duct without involving other tissues and the tumor completely blocking the bile duct lumen. Intraoperative frozen pathology showed no malignant tumor at the proximal cut end of the right and left hepatic duct and the distal cut end of the common bile duct. Roux-en-Y hepaticojejunostomy with excision of the extrahepatic biliary tree and radical lymphadenectomy were conducted. This procedure was considered curative since intraoperative frozen examination showed that the mass was mid atypia and the resection margin was negative. The jaundice was resolved completely and the patient was discharged 11 days after surgery without postoperative complications. The detailed postoperative pathological and immunohistochemical examination revealed a bile duct NET G1 with a size of 2 × 2 × 0.5 cm, and tumor cells infiltrated the all layers, CD56(+), Syn(+), CgA(+), CK20(−), CK7(−), Ki-67 < 2% (Fig. 3). Unexpectedly, a microscopic invasion of the resected right hepatic duct was observed in the final pathological examination. There is currently no criterion for biliary neuroendocrine tumors stage and no guideline for the treatment of biliary neuroendocrine tumors. These results were so complicated and we faced a challenge whether to continue to extend resection and adjuvant chemotherapy. After interdisciplinary discussion, we respected the patient's right to refuse second surgery and adjuvant chemotherapy was recommended. The patient received 3 cycles of adjuvant chemotherapy (Gemcitabine and tegafur-gimeracil-oteracil potassium capsule). The patient was followed up once a year for tumor markers and abdominal MRI. At present, the patient has been followed up for 24 months without recurrence or disease progression (Fig. 4).

## Discussion and conclusions

Preoperative diagnosis of EBNET is challenging because its lack of specific diagnostic indicators and extremely low incidence. Michalopoulos et al. [6] reported that preoperative diagnosis was only in 4 cases of the 150 EBNET cases between 1959 and 2012. The diagnosis of EBNET mainly relies on the postsurgical pathological and immunohistochemical examination. For this patient, the following questions were raised: (1) how should the surgeons make accurate preoperative diagnosis of EBNET? (2) Whether R1 resection of EBNET G1 requires further treatment and the therapeutic plan?

CgA can be elevated in both functional and non-functional NETs and can be a promising serum marker, but the sensitivity of CgA measurements in patient with NETs is only about 60–90% with a specificity of less than 50% [7]. Neuron-specific enolase (NSE) also has been utilized as a serum marker for NETs, and NSE can be elevated in 30–50% of NETs, especially in patients with high-grade tumors [7]. Functional NETs can produce some hormonal substances, such as somatostatin, polypeptides, serotonin, and calcitonin. It is useful to measure specific hormones in functional NETs. Both CA19-9 and CA-125 are commonly used serum tumor markers for the preoperative diagnosis of cholangiocarcinoma [8]. However, Wang et al. [9] reported that the positivity of CA19-9 was only 15.0%, CA72-4 was 7.5%, CEA was 17.5%, and AFP was 15.0% in primary hepatic neuroendocrine tumors. Furthermore, serum tumor markers are normal in most EBNET G1/G2, but they tend to be higher than normal levels in EBNET G3 or extrahepatic biliary neuroendocrine carcinomas [10–14]. Our patient with the hilar bile duct NETs G1 had serum tumor markers within normal limits.

Study [11] showed that the shape of EBNET could be divide into nodular, intraductal-growing, and periductal-infiltrating type. The imaging manifestations of enhanced CT in arterial-phase were as follows: (1) intraductal-growing type of EBNET indicated a higher density than the hepatic parenchyma, and this was helpful for distinguishing from the intraductal-growing type of cholangiocarcinoma showing a lower density than the hepatic parenchyma; (2) nodular type mainly showed equal density compared to hepatic parenchyma; (3) periductal-infiltrating type showed thickening of the bile duct wall and sudden blockage, which was similar to the distal bile duct cholangiocarcinoma. The imaging manifestations of MRI were as follows: (1) T1WI indicated that all tumors were lower SI than the hepatic parenchyma; (2) T2WI indicated that 80% of tumors were higher SI than the hepatic parenchyma; (3) DWI indicated that all tumors were higher SI than the hepatic parenchyma. The MRI manifestations of our patient were consistent with the study (Fig. 1).

Endoscopic and biopsy techniques can be used for preoperative diagnosis of EBNET. Lesions can be detected and biopsied by choledochoscopy, endoscopic retrograde cholangiopancreatography (ERCP), endoscopic ultrasound (EUS) and SpyGlass. Sano et al. [15] reported a case of well-differentiated EBNET diagnosed successfully by EUS-guided fine-needle aspiration biopsy. Besides, biliary brush cytology has been widely used in the preoperative diagnosis of biliary diseases, and the cytology of bile and bile duct brush specimens were also helpful for preoperative diagnosis of biliary NET [10, 16]. We regretted that the patient did not have bile juice cytology and step biopsy before surgery. Maybe bile juice cytology and step biopsy can help us make a correct preoperative diagnosis. The patient is very young, just 24 years old. This also reminds us that we should not easily diagnose cholangiocarcinoma in young patients, and more comprehensive preoperative examination is needed.

The unexpected pathological examination challenging us to give proper treatment. A multidisciplinary discussion proposed four options for this patient: (1) a second operation should be performed, and the entire tumor can be removed. But the new procedures would bring more surgical trauma to this patient. (2) The well-differentiated neuroendocrine cells are known to overexpress somatostatin receptors (SSR). The somatostatin analogs (SSA) or peptide receptor radionuclide therapy (PRRT) may be a good option for patients with well-differentiated NET G1. Martyn et al. [17] reported that compared with the placebo group, the SSA group could significantly prolong progression-free survival (PFS) in patients with metastatic enteropancreatic neuroendocrine tumors (Ki-67 < 10%). Ersin et al. [18] found that PFS in the SSA group was 21 months, which was better than the chemotherapy group as their first-line treatment for NET (Ki-67 ≤ 20%). PRRT has become an effective treatment for the NETs which express sufficient SSR. Both ^90^Y and ^177^Lu were used as radioactive isotopes in PRRT for NET. ^177^Lu–tetraazacyclododecanetetraacetic acid–octreotide (^177^Lu-DOTATATE) therapy was recommended for patients with SSR-positive NET in the US in January 2018 and in Europe in September 2017 [19]. To make sure SSR is positive, SSR imaging such as ^68^ Ga-DOTATATE PET/CT can be given firstly before PRRT is administered. Studies [20, 21] showed that ^68^ Ga-DOTATATE PET/CT was safer and efficient for diagnosis and treatment management of NET, and should be the preferred imaging method for preliminary diagnosis, selection of patients for PRRT, and localization of unknown primary tumors. (3) Systemic chemotherapy and targeted therapy may be a treatment choice for this patient, the chemotherapy can improve resectability and control tumor progression. However, chemotherapy is mainly used for patients with high-grade and metastatic NETs. And the role of chemotherapy is still undetermined in well-differentiated NETs, and still lacks standard indications [22]. Many targeted drugs are under research, but few targeted drugs have entered phase III clinical trials. At present, only sunitinib and everolimus are FDA-approved targeted drugs for NETs [23]. Targeted therapy gives hope for low-grade and intermediate-grade NETs. A phase III trial [24] showed that everolimus could significantly improve PFS for patients with advanced, well-differentiated, and non-functional NETs. However, there is no specific evidence to clarify that chemotherapy or targeted therapy can benefit the hilar bile duct NET G1 with R1 resection. (4) NET G1 may be considered indolent, no further treatment required, and requires regular follow-up.

Our patient chose no further therapy after three cycles of adjuvant chemotherapy and follow-up strictly at our outpatient service. At present, the patient has been following up for 24 months without recurrence or disease progression. Therefore, we believe that postoperative prophylactic intravenous chemotherapy is beneficial for NETs, especially for patients in G3. Many more clinical trials are ongoing, and these results will be clarified in the future. We know little of biliary NETs because of its rarity. There are currently no guidelines for the diagnosis and treatment of biliary NETs. Here we reported a case of perihilar bile duct NETs G1 with R1 resection, as far as we know this is the first report. More information about biliary NETs must be registered.

## Data Availability

The datasets used during the current study are available from the corresponding author on reasonable request.

## Abbreviations

NETs: Neuroendocrine tumors
EBNETs: Extrahepatic biliary neuroendocrine tumors
MRI: Magnetic resonance imaging
CEA: Carcinoembryonic antigen
AFP: Alpha-fetoprotein
CA: Carbohydrate antigen
CT: Computed tomography
T1WI: T1-weighted image
SI: Signal intensity
T2WI: T2-weighted image
DWI: Diffusion-weighted image
NSE: Neuron-specific enolase
SSR: Somatostatin receptors
SSA: Somatostatin analogs
PRRT: Peptide receptor radionuclide therapy
PFS: Progression-free survival
